# Angiopoietin-2 gene polymorphisms are biomarkers for the development and progression of colorectal cancer in Han Chinese

**DOI:** 10.7150/ijms.37675

**Published:** 2020-01-01

**Authors:** Zhang Du, Chih-Hsin Tang, Li-Jun Li, Le Kang, Jin Zhao, Lulu Jin, Chao-Qun Wang, Chen-Ming Su

**Affiliations:** 1 Department of Anorectal Surgery, Affiliated Dongyang Hospital of Wenzhou Medical University, Dongyang, Zhejiang, China.; 2 School of Medicine, China Medical University, Taichung, Taiwan.; 3 Chinese Medicine Research Center, China Medical University, Taichung, Taiwan.; 4 Department of Biotechnology, College of Health Science, Asia University, Taichung, Taiwan.; 5 Department of Biomedical Sciences Laboratory, Affiliated Dongyang Hospital of Wenzhou Medical University, Dongyang, Zhejiang, China.; 6 Department of Pathology, Affiliated Dongyang Hospital of Wenzhou Medical University, Dongyang, Zhejiang, China.; 7 Department of Sports Medicine, China Medical University, Taichung, Taiwan.

**Keywords:** Angiopoietin-2, colorectal cancer, polymorphism

## Abstract

Colorectal cancer (CRC) is one of the most common cancers in Han Chinese and is characterized by low rates of early diagnosis and poor survival rates. Angiopoietin-2 (Ang2), an endothelial tyrosine kinase, is involved in CRC progression, but little is known about the association between single nucleotide polymorphisms (SNPs) and diagnosis or prognosis of CRC. This study reports on the association between 5 SNPs of the *Angpt2* gene (rs2442598, rs734701, rs1823375, 11137037, and rs12674822) and CRC susceptibility as well as clinical outcomes in 379 patients with CRC and in 1,043 cancer-free healthy controls. Carriers of the CG allele at rs1823375 and those with the GT+TT allele of the variant rs12674822 were at greater risk of CRC than their respective wild-type counterparts. Moreover, carriers of the GT or GT+TT allele in rs12674822 were significantly more likely to have tumor involvement in both the colon and rectum compared with wild-type (GG) carriers, while 5-year progression-free survival was also significantly worse in those carrying the GT+TT allele in rs12674822 compared with wild-type carriers. Our study is the first to describe correlations between *Angpt2* polymorphisms and CRC development and progression in people of Chinese Han ethnicity.

## Introduction

Epidemiologic evidence demonstrates a rapid increase in the incidence of CRC in China since the mid-2000s, which has been attributed to changes in lifestyle and dietary behaviors, including an increasingly sedentary lifestyle, a Westernized diet and increasing alcohol consumption 1, 2. Colonoscopy is considered to be the gold standard for CRC investigation 3, with proven efficacy in CRC prevention 4-6. However, the ultimate efficacy of CRC screening programs depends upon the technique and technology of colonoscopy for accurately detecting colorectal neoplasia and CRC prevention; the protective effect of this procedure is severely compromised by evidence showing a wide variability in endoscopist performance in terms of adenoma detection rate, a proxy for the accuracy of the endoscopist 7. Thus, the clinical impact of colonoscopy remains uncertain.

Moreover, current diagnostic methods for CRC detection are limited by various features such as the risk of tearing or perforation (i.e., colonoscopy, flexible sigmoidoscopy), or the failure to the failure to detect most polyps and some cancers (i.e., fecal blood tests) and by the fact that CRC is largely asymptomatic until it progresses to the advanced stages 8. Diagnosing cytogenetic alterations could serve as more accurate noninvasive biomarker detection methods that provide diagnostic, prognostic and predictive CRC molecular markers in blood and stool 8. CRC is associated with single nucleotide polymorphisms (SNPs) in gene loci, so analyses of the correlation between clinical status and SNP genotyping data can serve as a strategy for improving the diagnosis of CRC, predicting the risk of developing CRC and personalized treatment protocols 9-11. SNPs in angiopoietin-2 (Angpt2) have been investigated for their association with lung and breast cancers 12, 13. *Angpt2* is localized to the human chromosome band 8p23.1 and supports endothelial cell homeostasis in terms of its angiogenic function 14, 15. Wang and colleagues observed a significant positive correlation between levels of connective tissue growth factor (CTGF) and *Angpt2* expression and similarly between CTGF and *Angpt2* mRNA expression, as well as a significant association between high levels of *Angpt2* expression and both clinical stage and metastasis in patients with osteosarcoma 16. In another investigation, two SNPs in *Angpt2* (rs1868554 and rs2442598) were significantly associated with acute lung injury in clinical stage II 13, while another study reported an association between five *Angpt2* variants (s2442598, rs734701, rs1823375, rs11137037 and rs1267482) and associated with the risk of developing rheumatoid arthritis 17. So far, no studies have searched for correlations between *Angpt2* gene polymorphisms and CRC susceptibility amongst Han Chinese. We therefore performed a case-control study to evaluate the possible involvement of SNPs in the *Angpt2* gene and CRC susceptibility in a cohort of Han Chinese patients diagnosed with CRC.

## Materials and Methods

### Study participants and blood samples

We collected blood specimens from 379 patients (cases) diagnosed with CRC in Dongyang People's Hospital between January 1, 2014 and August 31, 2018. A total of 1,043 healthy subjects without any history of cancer were also enrolled into the study and served as the control group. All study participants provided written informed consent for the study, which was approved by the Ethics Committee of Dongyang People's Hospital and the hospital's Institutional Review Board. Clinicopathologic characteristics were determined for all patients based on their medical records and all study participants completed a standardized questionnaire that acquired detailed clinical data on age, sex, smoking history and levels of alcohol consumption. Whole blood samples (3 mL) were collected from all study participants and stored at -80°C for subsequent DNA extraction.

### Selection of *Angpt2* polymorphisms

Five *Angpt2* SNPs (rs2442598, rs734701, rs1823375, 11137037 and rs12674822) were selected from the intron of *Angpt2*; all SNPs had minor allele frequencies of greater than 5%. Most *Angpt2* SNPs were known to be associated with lung injury or breast cancer 13, 18.

### Genomic DNA extraction

Genomic DNA was extracted from peripheral blood leukocytes using a QIAamp DNA blood kit (Qiagen, CA, USA) according to the manufacturer's instructions. Extracted DNA was stored at -20°C and prepared for genotyping by polymerase chain reaction (PCR).

### Genotyping by real-time PCR

Total genomic DNA was isolated from whole blood specimens using QIAamp DNA blood mini kits (Qiagen, Valencia, CA), as per the manufacturer's instructions. DNA was dissolved in TE buffer (10 mM Tris pH 7.8, 1 mM EDTA) and stored at -20°C until quantitative PCR analysis. Five *Angpt2* SNP probes were purchased from Thermo Fisher Scientific Inc. (USA) and assessment of allelic discrimination for *Angpt2* SNPs was conducted using a QuantStudio^TM^ 5 Real-Time PCR system (Applied Biosystems, CA, USA), according to the manufacturer's instructions. Data were further analyzed with QuantStudio™ Design & Analysis Software (Applied Biosystems) and analytic statistics were compiled with clinical data 19. PCR genotyping was performed in a total volume of 10 μL, containing 20-70 ng genomic DNA, 1 U Taqman Genotyping Master Mix (Applied Biosystems, Foster City, CA, USA), and 0.25 μL probes. The protocol included an initial denaturation step at 95°C for 10 min, followed by 40 cycles of 95°C for 15 s and 60°C for 1 min.

### Statistical analysis

Between-group differences were considered significant if p values were less than 0.05. Hardy-Weinberg equilibrium (HWE) was assessed using the Chi-square goodness-of-fit test for biallelic markers. Mann-Whitney *U*-tests and the Fisher's exact test were used to compare differences in demographic characteristics between healthy controls and patients with CRC. The odds ratios (ORs), adjusted odds ratios (AORs) and 95% confidence intervals (CIs) for associations between genotype frequencies and the risk of CRC or clinicopathologic characteristics were estimated by multiple logistic regression analysis that controlled for age, cigarette and alcohol consumption. Since the data was independent and normal distribution, Fisher's exact test was used to compare differences in demographic characteristics between healthy controls and patients with CRC and Bonferroni's correction for multiple comparisons.

Cox proportional hazards regression modeling evaluated the effects of *Angpt2* genotyping results on the probability of recurrence or overall survival (OS) in multivariate analysis that adjusted for age. OS, including 5-year survival rate or probability of recurrence by the end of the study (August, 2018), was analyzed using Kaplan-Meier and univariate and multivariate Cox regression modeling. All data were analyzed using Statistical Analytic System software (v. 9.1, 2005; SAS Institute, Cary, NC, USA).

## Results

We recruited 379 CRC patients and 1,043 cancer-free healthy subjects, all of whom were of Han Chinese ethnicity. Demographic and clinicopathologic characteristics are described in Table 1. The mean age was 40.47 ± 15.61 years for the patients with CRC (cases) and 53.07 ± 11.31 years for the controls (*p* < 0.05). Both cohorts were significantly more likely to be non-smokers and less likely to consume alcohol (both *p* < 0.05). As according to the American Joint Committee on Cancer (AJCC) TNM staging system (6^th^ edition), the CRC patients were defined as clinical stages I+II (49.1%) or III+IV (50.9%). They were also divided into two groups in terms of their tumor site; i.e., the colon (58.6%) or rectum (41.4%) (Table 1).

Genotyping analysis of cases and controls evaluated associations between the five *Angpt2* SNPs (rs2442598, rs734701, rs1823375, 11137037 and rs12674822) and the risk of CRC; the results are given in Table 2. The alleles with the highest distribution frequency at *Angpt2* for rs2442598, rs734701, rs1823375, 11137037 and rs12674822 in both cases and controls were heterozygous AT, heterozygous TC, homozygous CC, homozygous AA and heterozygous GT, respectively (Table 2). Individuals carrying CG at rs1823375 had a 1.340-fold (95% CI: 1.002-1.74, *p* < 0.05) higher risk of CRC compared with individuals carrying the wild-type CC polymorphic allele, in analyses adjusting for age. Having the GT and GT+TT genotypes of SNP rs12674822 significantly increased the risk of developing CRC (OR: 1.419, 95% CI: 1.063-1.892; OR: 1.403, 95% CI: 1.069-1.842, respectively, both *p* < 0.05), compared with having the GG genotype (Table 2).

Next, *Angpt2* genotypes in patients with CRC were investigated to clarify the risk of *Angpt2* polymorphisms as according to clinical TNM stage, tumor site and pathologic grade. For this cohort, our initial analysis of the SNP rs12674822 revealed a significant correlation between rs12674822 variants (GG vs GT and GT+TT, respectively) and tumor sites (OR: 1.867, 95% CI: 1.101-3.168; OR: 1.746, 95% CI: 1.055-2.889, respectively; *p* < 0.05 for both comparisons) (Table 3). No significant findings were observed between other *Angpt2* genotypes and clinicopathologic status (data not shown). In addition, no significant patterns of linkage disequilibrium were observed in any of the *Angpt2* genotypes analyzed from CRC patients (data not shown).

**Figure 1:**
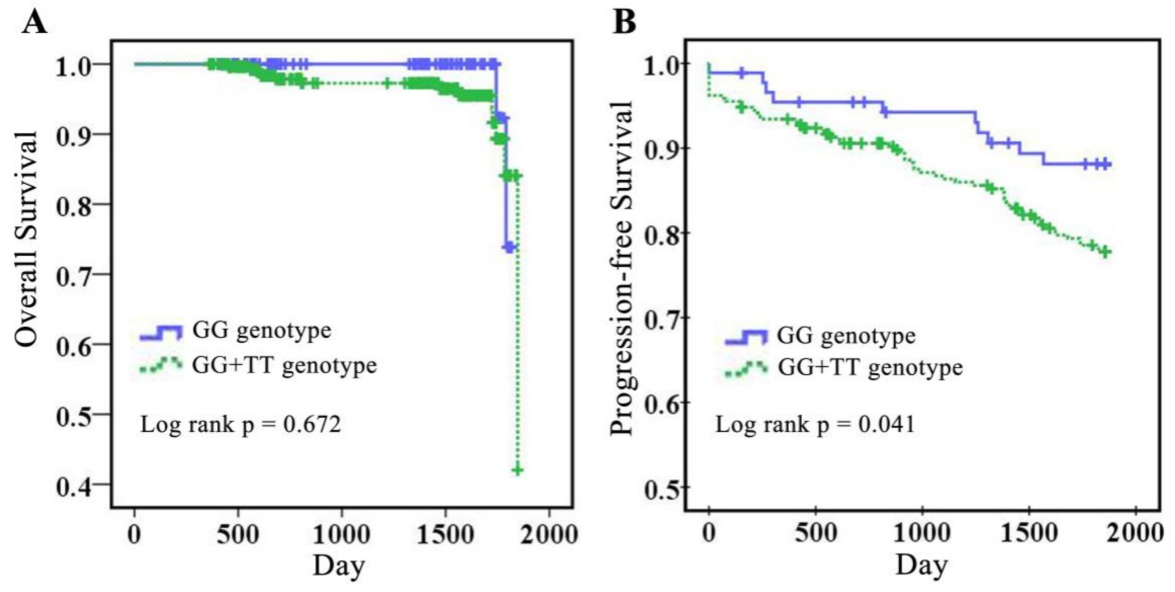
** Association of *Angpt2* rs12674822 with five-year survival in 379 colorectal cancer patients.** (A) Kaplan-Meier analysis of the correlation between rs12674822 genotypes and overall survival in 379 colorectal cancer patients. (B) Kaplan-Meier analysis of the correlation between the rs12674822 genotype and progression-free survival in colorectal cancer patients.

*Angpt2* is known to be involved in CRC progression and survival 20. We used Kaplan-Meier survival analysis to demonstrate the correlation of *Angpt2* polymorphisms with OS and progression-free survival (PFS) (Fig. 1). Interestingly, CRC patients carrying GT+TT at rs12674822 had lower five-year OS (*p* = 0.672) and significantly worse PFS (*p* = 0.041), compared to those with the GG genotype. This finding suggests that presence of the *Angpt2* polymorphism at rs12674822 could serve as a genetic marker for PFS in Han Chinese patients with CRC.

## Discussion

The main objective of this hospital-based, case-control study was to investigate the association between *Angpt2* polymorphisms and CRC susceptibility in a Han Chinese population. We investigated the roles of rs2442598, rs734701, rs1823375, 11137037 and rs12674822 polymorphisms of the *Angpt2* gene on the risk of developing CRC and on five-year survival of the CRC cohort. Our results revealed that genetic variants of the *Angpt2* rs12674822 polymorphism appears to be correlated with the risk of CRC and PFS in CRC patients. To the best of our knowledge, our study is the first to provide evidence on associations between *Angpt2* polymorphisms and CRC susceptibility in a Han Chinese population.

As with vascular endothelial growth factor (VEGF), the role of *Angpt2* has been characterized as being pro-angiogenic or pro-inflammatory in cancer progression 21-24. However, there are few reports regarding *Angpt2* polymorphisms in various types of cancers. An American study that evaluated genetic associations between 17 candidate genes, including *Angpt2*, and the development of lymphedema following treatment for breast cancer concluded that *Ang2* rs1823375 has no significant involvement in lymphangiogenesis or angiogenesis 18. Previous studies have reported that *Angpt2* gene polymorphisms are associated with sleep-disordered breathing 25 and lung injury syndrome 13, 26, 27, indicating that *Angpt2* could be a biomarker for lung disease. Another study, in an African American cohort, has reported that two SNPs in *Angpt2* (rs1868554 and rs2442598) are significantly associated with acute lung injury pathogenesis and susceptibility 13. Our recent study also demonstrated that the *Angpt2* rs11137037 polymorphism was associated with a high risk of clinical-stage lung cancer in people of Han Chinese ethnicity 28. A recent study has revealed that *Angpt2* polymorphisms were associated with patients with resected colorectal liver metastases 29. In this study, we extended our numbers of patients and controls from our previous study 30-32. Our results reveal that smoking and alcohol consumption are significant risk factors for CRC (Table 1; *p* < 0.05 for both comparisons). Specifically, *Angpt2* rs1823375 and rs12674822 polymorphisms were associated with a higher susceptibility for CRC, in analyses controlling for smoking and alcohol consumption.

A systematic review has demonstrated that tumor-infiltrating lymphocytes have prognostic and predictive significance in colon, rectal and metastatic CRC 33. Our results indicate that the GT and GT+TT variants of the SNP rs12674822 are significantly correlated with tumor site (i.e., the rectum). Our linkage disequilibrium analysis of all *Angpt2* SNPs revealed no specific results (Supplementary Figure S1). The highest risk genotypes were rs734701 TC and rs2442598 AT (*r^2^* = 0.66) (data not shown). Linkage disequilibrium between *Angpt2* rs1823375 CG and *Angpt2* rs12674822 GT (*r^2^* = 0.12) had no such association, suggesting that it requires further analysis in other CRC cohorts.

In this study, we found that *Angpt2* rs12674822 GT+TT were significantly related to PFS in CRC patients. SNP rs12674822 is located in the intron of *Angpt2* and *MCPH1*, which each induce endothelial cell apoptosis and cell cycle arrest, respectively 34-36. We propose that rs12674822 may increase *Angpt2* mRNA expression by affecting tumor-derived endothelial cell apoptosis in CRC. Further investigations are needed to elucidate this aspect.

Limitations of this study include common issues such as the small sample size. Further studies with larger patient populations should explore the association between *Ang2* and CRC susceptibility. Second, our study was restricted to a single Han Chinese cohort, which calls for further analyses of different Han Chinese cohorts, especially with regard to Western-style dietary consumption. Third, *KRAS* and *BRAF* mutation data were unavailable for the CRC cohort, which might be due to the fact that such examinations are expensive in China and that the diagnostic procedure there are complicated by long waiting times between clinic appointments and expenses relating to time off work, as well as the diagnostic procedures themselves.

Our results demonstrate associations between *Angpt2* rs1823375 and rs12674822 polymorphisms and risk of CRC; our evidence shows a significant association between *Angpt2* rs12674822 and tumor site in a Han Chinese cohort. We also demonstrate a correlation between SNP rs12674822 and PFS in CRC patients. This study is the first to report a correlation between *Angpt2* polymorphisms and CRC risk. Thus, *Angpt2* could be developed as a genetic prognostic marker for CRC prognosis and prediction.

## Supplementary Material

Supplementary figures.

## Figures and Tables

**Table 1:** Demographic and clinicopathologic characteristics of healthy controls and patients with colorectal cancer

Variable	Controls N=1,043 (%)	Patients N=379 (%)	*p* value
**Age (years)**	Mean ± S.D.	Mean ± S.D.	
	40.47 ± 15.61	53.07 ± 11.31	*p* < 0.05
**Gender**			
Female	539 (51.7)	169 (44.6)	*p* < 0.05
Male	504 (48.3)	210 (55.4)	
**Cigarette smoking**			
No	928 (89.0)	273 (72.0)	*p* < 0.05
Yes	115 (11.0)	106 (28.0)	
**Alcohol consumption**			
No	907 (87.0)	258 (68.1)	*p* < 0.05
Yes	136 (13.0)	121 (31.9)	
**Clinical stage**			
I+II		186 (49.1)	
III+IV		193 (50.9)	
**Tumor size**			
≤T2		70 (18.5)	
>T2		309 (81.5)	
**Lymph node status**			
N0		187 (49.3)	
N1+N2+N3		192 (50.7)	
**Distant metastasis**			
M0		351 (92.6)	
M1		28 (7.4)	
**Histological grade**			
Gx+G1		89 (23.5)	
G2+G3		290 (76.5)	
**Tumor site**			
Colon		157 (58.6)	
Rectum		222 (41.4)	

The Mann-Whitney *U*-test or Fisher's exact test was used to compare values between controls and patients with colorectal cancer. * *p* < 0.05 was considered to be statistically significant.

**Table 2:** Distribution frequencies of *Angpt2* genotypes in controls and patients with colorectal cancer

Variable	Controls N=1043 (%)	Patients N=379 (%)	OR (95% CI)	AOR (95% CI)
**rs2442598**				
AA	275 (26.4)	100 (26.4)	1.00 (reference)	1.00 (reference)
AT	544 (52.2)	183 (48.5)	0.925 (0.697─1.228)	0.935 (0.667─1.309)
TT	224 (21.5)	96 (25.3)	1.179 (0.846─1.641)	1.224 (0.825─1.815)
AT+TT	648 (72.0)	507 (73.0)	0.999 (0.765─1.304)	1.021 (0.745─1.399)
**rs734701**				
TT	353 (33.8)	113 (29.8)	1.00 (reference)	1.00 (reference)
TC	476 (45.6)	195 (51.5)	1.280 (0.977─1.676)	1.297 (0.948─1.773)
CC	214 (20.5)	71 (18.7)	1.036 (0.736─1.459)	1.099 (0.730─1.654)
TC+CC	595 (66.1)	469 (67.5)	1.204 (0.933─1.554)	1.228 (0.911─1.656)
**rs1823375**				
CC	516 (49.5)	172 (45.4)	1.00 (reference)	1.00 (reference)
CG	442 (42.4)	173 (45.6)	1.174 (0.918─1.502)	1.340 (1.002─1.794)*
GG	85 (8.1)	34 (9.0)	1.200 (0.778─1.851)	1.082 (0.646─1.810)
CG+GG	449 (49.9)	353 (50.8)	1.178 (0.931─1.492)	1.293 (0.979─1.708)
**rs11137037**				
AA	491 (47.1)	188 (49.6)	1.00 (reference)	1.00 (reference)
AC	392 (37.6)	123 (32.5)	0.819 (0.630─1.066)	0.953 (0.701─1.296)
CC	160 (15.3)	68 (17.9)	1.110 (0.798─1.544)	1.200 (0.806─1.786)
AC+CC	483 (53.7)	351 (50.5)	0.904 (0.714─1.143)	1.030 (0.780─1.359)
**rs12674822**				
GG	314 (30.1)	89 (27.5)	1.00 (reference)	1.00 (reference)
GT	485 (46.5)	352 (50.6)	1.419 (1.063─1.892)*	1.443 (1.025─2.031)*
TT	244 (23.4)	152 (21.9)	1.374 (0.984─1.918)	1.306 (0.882─1.934)
GT+TT	617 (68.6)	504 (72.5)	1.403 (1.069─1.842)*	1.384 (1.006─1.904)*

The odds ratios (ORs) and their associated 95% confidence intervals (CIs) were estimated by logistic regression analysis. The adjusted odds ratios (AORs) with their associated 95% CIs were estimated by multiple logistic regression analysis that controlled for smoking, alcohol consumption and age. * *p* < 0.05 was considered to be statistically significant.

**Table 3:** Odds ratios (ORs) and 95% confidence intervals (CIs) of clinical status and *Angpt2* rs12674822 genotypic frequency in 379 patients with colorectal cancer.

Genotypes	Patients		OR (95% CI)	AOR (95% CI)
**Clinical Stage**				
	**Stage I+II**	**Stage III+IV**		
**rs12674822**	**N=186 (%)**	**N=193 (%)**		
GG	51 (27.4)	38 (19.7)	1.00 (reference)	1.00 (reference)
GT	89 (47.8)	106 (54.9)	1.598 (0.964─2.651)	1.599 (0.964─2.651)
TT	46 (24.7)	49 (25.4)	1.430 (0.799─2.558)	1.441 (0.804─2.584)
GT+TT	135 (72.6)	155 (80.3)	1.541 (0.954─2.488)	1.542 (0.955─2.491)
**Tumor size**				
	**≤ T2**	**> T2**		
**rs12674822**	**N=70 (%)**	**N=309 (%)**		
GG	16 (22.9)	73 (23.6)	1.00 (reference)	1.00 (reference)
GT	33 (47.1)	162 (52.4)	1.076 (0.557─2.077)	1.077 (0.557─2.083)
TT	21 (30.0)	74 (23.9)	0.772 (0.374─1.597)	0.768 (0.371─1.590)
GT+TT	54 (77.1)	236 (76.4)	0.958 (0.517─1.775)	0.958 (0.517─1.776)
**Lymph node metastasis**				
	**N0**	**N1+N2+N3**		
**rs12674822**	**N=187 (%)**	**N=192 (%)**		
GG	51 (27.3)	38 (19.8)	1.00 (reference)	1.00 (reference)
GT	90 (48.1)	105 (54.7)	1.566 (0.944─2.596)	1.566 (0.944─2.596)
TT	46 (24.6)	49 (25.5)	1.430 (0.799─2.558)	1.441 (0.804─2.584)
GT+TT	136 (72.7)	154 (80.2)	1.520 (0.941─2.454)	1.521 (0.942─2.457)
**Distant metastasis**				
	**M0**	**M1**		
**rs12674822**	**N=351 (%)**	**N=28 (%)**		
GG	84 (23.9)	5 (17.9)	1.00 (reference)	1.00 (reference)
GT	179 (51.0)	16 (57.1)	1.502 (0.532─4.236)	1.501 (0.532─4.238)
TT	88 (25.1)	7 (25.0)	1.336 (0.408─4.375)	1.325 (0.404─4.354)
GT+TT	267 (76.1)	23 (82.1)	1.447 (0.534─3.925)	1.444 (0.532─3.923)
**Tumor sites**				
	**Colon**	**Rectum**		
**rs12674822**	**N=222 (%)**	**N=157 (%)**		
GG	61 (27.5)	28 (17.8)	1.00 (reference)	1.00 (reference)
GT	105 (47.3)	90 (57.3)	1.867 (1.101─3.168)*	1.876 (1.104─3.189)*
TT	56 (25.2)	39 (24.8)	1.517 (0.828─2.781)	1.520 (0.829─2.788)
GT+TT	161 (72.5)	129 (82.2)	1.746 (1.055─2.889)*	1.751 (1.057─2.900)*
**Pathologic grade**				
	**Gx+G1**	**G2+G3**		
**rs12674822**	**N=36 (%)**	**N=290 (%)**		
GG	9 (25.0)	69 (23.8)	1.00 (reference)	1.00 (reference)
GT	17 (47.2)	153 (52.8)	1.174 (0.499─2.764)	1.174 (0.498─2.764)
TT	10 (27.8)	68 (23.4)	0.887 (0.339─2.318)	0.880 (0.336─2.305)
GT+TT	27 (75.0)	221 (76.2)	1.068 (0.479─2.379)	1.065 (0.478─2.375)

The ORs with their 95% CIs were estimated by logistic regression models. The adjusted odds ratios (AORs) with their 95% CIs were estimated by multiple logistic regression models that controlled for age. * *p* < 0.05 was considered to be statistically significant.
