# Supplementary material for: Angiopoietin-2 gene polymorphisms are biomarkers for the development and progression of colorectal cancer in Han Chinese
Source: Int J Med Sci. 2020 Jan 1;17(1):97–102. doi: 10.7150/ijms.37675 (PMC6945552; doi:10.7150/ijms.37675)

Angiopoietin-2 gene polymorphisms are biomarkers for the development and progression of colorectal cancer in Han Chinese

Supplementary Figure S1

Fig. S1. Linkage disequilibrium patterns of five single nucleotide polymorphisms in the *Ang2* gene.

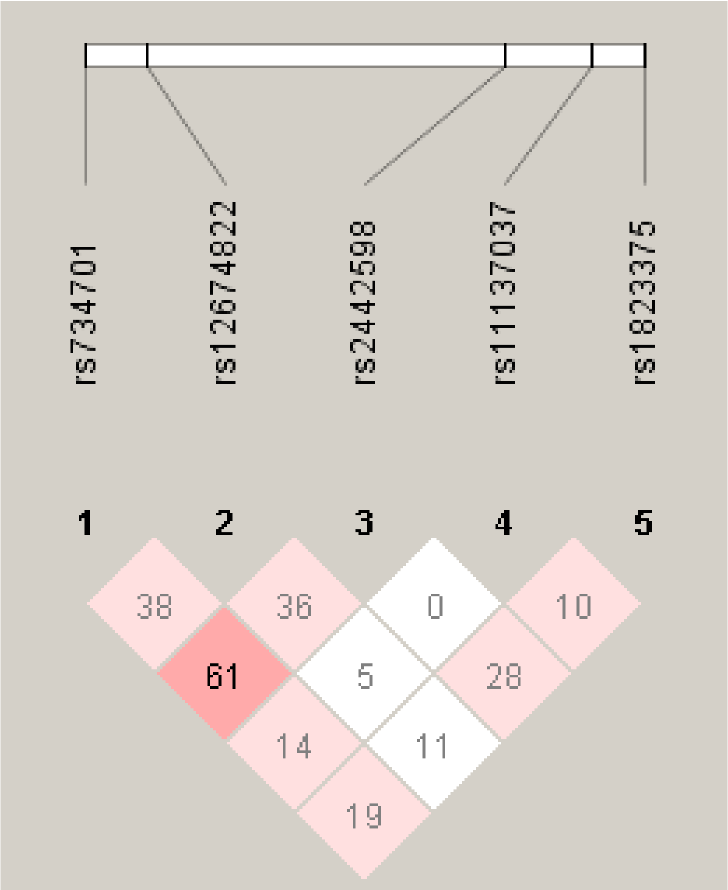

Supplement: Supplementary file 1 — Supplementary figures. [file ijmsv17p0097s1.pdf]
